# Rapid and Repetitive Inactivation of SARS‐CoV‐2 and Human Coronavirus on Self‐Disinfecting Anionic Polymers

**DOI:** 10.1002/advs.202003503

**Published:** 2021-03-08

**Authors:** Bharadwaja S. T. Peddinti, Sierra N. Downs, Jiaqi Yan, Steven D. Smith, Reza A. Ghiladi, Vijay Mhetar, Roger Tocchetto, Anthony Griffiths, Frank Scholle, Richard J. Spontak

**Affiliations:** ^1^ Department of Chemical & Biomolecular Engineering North Carolina State University Raleigh NC 27695 USA; ^2^ National Emerging Infectious Diseases Laboratories Boston University School of Medicine Boston MA 02118 USA; ^3^ Corporate Research & Development The Procter & Gamble Company Cincinnati OH 45224 USA; ^4^ Department of Chemistry North Carolina State University Raleigh NC 27695 USA; ^5^ Center for Advanced Virus Experimentation North Carolina State University Raleigh NC 27695 USA; ^6^ Kraton Innovation Center Kraton Corporation Houston TX 77084 USA; ^7^ Department of Biological Sciences North Carolina State University Raleigh NC 27695 USA; ^8^ Department of Materials Science & Engineering North Carolina State University Raleigh NC 27695 USA

**Keywords:** antiviral materials, charged block polymers, microphase‐ordered materials, polyanions, thermoplastic elastomers

## Abstract

While the ongoing COVID‐19 pandemic affirms an urgent global need for effective vaccines as second and third infection waves are spreading worldwide and generating new mutant virus strains, it has also revealed the importance of mitigating the transmission of SARS‐CoV‐2 through the introduction of restrictive social practices. Here, it is demonstrated that an architecturally‐ and chemically‐diverse family of nanostructured anionic polymers yield a rapid and continuous disinfecting alternative to inactivate coronaviruses and prevent their transmission from contact with contaminated surfaces. Operating on a dramatic pH‐drop mechanism along the polymer/pathogen interface, polymers of this archetype inactivate the SARS‐CoV‐2 virus, as well as a human coronavirus surrogate (HCoV‐229E), to the minimum detection limit within minutes. Application of these anionic polymers to frequently touched surfaces in medical, educational, and public‐transportation facilities, or personal protection equipment, can provide rapid and repetitive protection without detrimental health or environmental complications.

## Graphical abstract

In the presence of water, the low pH that develops on nanostructured anionic block polymers prepared as stand‐alone films or surface coatings successfully inactivates pathogenic coronaviruses such as SARS‐CoV‐2 by at least 99.9% in just a few minutes. This class of sulfonated materials serves as a new and expedient route to combat the transmission of disease.

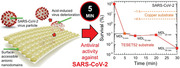

As an ongoing example of a catastrophic global health crisis, the COVID‐19 pandemic caused by the SARS‐CoV‐2 virus has pummeled strong national economies,^[^ ^1^ ^]^ imposed unprecedented social restrictions,^[^ ^2^ ^]^ and above all else, claimed over 2.3 million lives worldwide (with over 460 000 in the U.S. and over 560 000 in Europe) at the time of this publication.^[^ ^3^ ^]^ Although the enveloped SARS‐CoV‐2 virus is classified as a respiratory pathogen due to its ability to compromise the function of, as well as damage, the lungs,^[^ ^4^ ^]^ it is linked to an increased risk of health complications in adults with cardiopulmonary disease.^[^ ^5^ ^]^ A Kawasaki‐like disease has also been reported^[^ ^6^ ^]^ in children infected with SARS‐CoV‐2. The virus is primarily transmitted via miniscule droplets or aerosols^[^ ^7^ ^]^ that disperse in the air during speaking, coughing, or sneezing, thereby necessitating the wearing of facemasks and other protective personal equipment (PPE). Traditionally, these fibrous products function by excluding transmission of particles to body entry sites on the sole basis of size.^[^ ^8^ ^]^ Another route by which SARS‐CoV‐2 transmits, however, is via indirect transmission by contact with contaminated surfaces.^[^ ^9^ ^]^ For instance, Munster and co‐workers^[^ ^10^ ^]^ have observed that the virus can remain stable on surfaces for long periods of time: up to 2–3 days on stainless steel and unspecified plastic at 21–23 °C and 40% relative humidity. Chin et al.^[^ ^11^ ^]^ have measured longer surface stability times, such as 7 days on surgical masks, which highlights why disposable PPE should not be reused. Moreover, the survivability of this virus on different surfaces appears to be temperature‐dependent: infectious virus measured at or above the limit of detection has recently been reported^[^ ^12^ ^]^ for commonly encountered surfaces (including paper and plastic banknotes) over the course of a few days at 40 °C to nearly a month at 20 °C. These results confirm that SARS‐CoV‐2, as well as other coronaviruses,^[^ ^13^ ^]^ are capable of spreading after contact with a contaminated surface, which is worrisome in light of post‐reopening surges and the possibilities of mutations that increase infectivity^[^ ^14^ ^]^ or a more severe wave in the future.

Several approved vaccines are currently available to protect the global populace from the deleterious effects of SARS‐CoV‐2, but vaccine distribution requires adequate time and infrastructure to ensure sufficient public safety. While vaccines are presently being distributed to the populace, additional measures that are capable of affording rapid and continuous prevention of SARS‐CoV‐2 proliferation are clearly warranted to help mitigate its worldwide circulation. Whereas various chemical and radiative disinfecting protocols have been approved for this purpose, they require repeated application and are only virucidal during application (i.e., they do not prevent re‐contamination). In addition, the continual use of approved disinfectants containing, for example, quaternary ammonium compounds can negatively impact the environment.^[^ ^15^ ^]^ A more strategic approach to prevention involves the development of self‐disinfecting materials that can continuously inactivate the SARS‐CoV‐2 virus. Although examples of antibacterial materials are relatively common and frequently rely on the incorporation of metals^[^ ^16^ ^]^ (e.g., silver or copper) or metal oxides^[^ ^17^ ^]^ (e.g., zinc oxide), these bactericidal approaches suffer from known shortcomings, including gradual loss of germicidal activity^[^ ^18^ ^]^ and leaching‐induced environmental contamination,^[^ ^19^ ^]^ and are generally not as effective against viruses. Other antibacterial materials utilize cationic polymers as free‐standing films, coatings, or surface grafts.^[^ ^20^ ^]^ Alternatively, comprehensive effectiveness against several bacteria (both Gram‐positive and Gram‐negative, as well as antibiotic‐resistant), as well as enveloped/non‐enveloped viruses, has been achieved^[^ ^21^, ^22^ ^]^ in polymeric materials modified with photosensitive dye molecules that are capable of generating singlet oxygen in the presence of molecular oxygen and noncoherent visible light. These self‐disinfecting materials can inactivate a broad spectrum of infectious microbes after initial contact, and they remain continuously antimicrobial over extended periods of time.

We recently reported^[^ ^23^ ^]^ a highly effective and fast‐acting approach to self‐disinfecting materials based on the use of anionic block polymers. This class of materials consists of long sequences of dissimilar chemical species wherein one species is negatively charged. Because of their inherent thermodynamic incompatibility, polymers of this archetype typically self‐assemble into soft nanostructures.^[^ ^24^ ^]^ Due to the presence of sulfonic acid groups along the polymer backbone, these materials fully inactivate (99.9999%, 6 log_10_ units) a wide range of Gram‐positive and Gram‐negative bacteria (including methicillin‐resistant *Staphylococcus aureus*, MRSA) typically responsible for nosocomial infections, as well as enveloped/non‐enveloped viruses (including influenza A), after exposure at ambient temperature for just 5 min.^[^ ^23^ ^]^ The mechanism by which inactivation proceeds derives from a dramatic pH drop at the polymer/pathogen interface and depends on the number of sulfonic acid moieties present on each polymer molecule (expressed as the degree of sulfonation, DOS). Prior results reveal that the DOS affects the kinetics of inactivation, thereby indicating that a critical DOS level is required to achieve rapid and substantial microbial inactivation. In this work, we explore the time‐dependent inactivation of coronaviruses deposited on a family of anionic block polymers. To do so safely, we examine the less virulent human coronavirus (HCoV‐229E, a temperature‐sensitive^[^ ^25^ ^]^ common cold virus that can survive on various surfaces^[^ ^26^ ^]^ for 2–6 days) as a surrogate for SARS‐CoV‐2 on several block polymers differing in molecular architecture and chemical modification to demonstrate broad activity across this class of polymers, as well as provide mechanistic insight into the structure‐function properties associated with virucidal efficacy. In addition, we further investigate the time‐dependent inactivation of the SARS‐CoV‐2 virus on one particularly promising anionic block polymer that, as a thermoplastic elastomer, likewise possesses favorable mechanical properties. For further comparison, the antiviral properties of a chemically‐ and architecturally‐dissimilar perfluorinated polyeletrolyte (Nafion), composed of 1,1,2,2‐tetrafluoro‐2‐({1,1,1,2,3,3‐hexafluoro‐3‐[(trifluorovinyl)oxy]‐2‐propanyl}‐oxy)ethanesulfonic acid and tetrafluoroethylene, are also analyzed here.

## Experimental Section

**Scheme 1:**
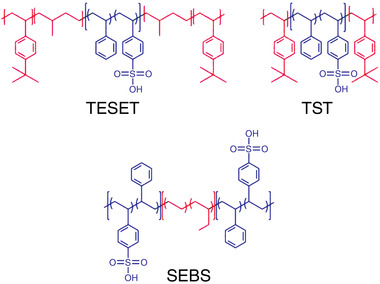
Chemical structures of the three classes of anionic block polymers investigated here: TESET, TST, and SEBS (labeled and defined in the Experimental Section). Hydrophilic and hydrophobic moieties are colored blue and red, respectively.

Three types of anionic block polymers were employed as virucidal substrates in this study, and their chemical structures are presented in **Scheme** **1**, wherein the color scheme differentiates the hydrophilic (blue) and hydrophobic (red) segments. Only a poly[*tert*‐butylstyrene‐*b*‐(ethylene‐*alt*‐propylene)‐*b*‐(styrene‐*co*‐styrenesulfonate)‐*b*‐(ethylene‐*alt*‐propylene)‐*tert*‐butylstyrene] (TESET) block polymer (available as BIAXAM^TM^ from Kraton Corporation) was used to investigate the survival kinetics of the SARS‐CoV‐2 virus at ambient temperature. The two other sulfonated block polymers depicted in Scheme 1, poly[*tert*‐butylstyrene‐*b*‐(styrene‐*co*‐styrenesulfonate)‐*b*‐*tert*‐butylstyrene] (TST) and poly[(styrene‐*co*‐styrenesulfonate)‐*b*‐(ethylene‐*co*‐butylene)‐*b*‐[(styrene‐*co*‐styrenesulfonate)] (SEBS), were selected here to study the survival kinetics of the HCoV‐229E surrogate and determine differences due to polymer architecture. These two pre‐sulfonated materials were synthesized in‐house (TST) or provided commercially (SEBS, Kraton Corporation) and subsequently sulfonated to different DOS levels. The number‐ and weight‐average molecular weights (*M*_n_ and *M*_w_), as well as the composition (expressed as a weight percent of the *i*th block, *w*_*i*_), of each pre‐sulfonated block polymer were measured by size‐exclusion chromatography and proton nuclear magnetic resonance spectroscopy. The results are listed in **Table** **1**, and the dispersity (Đ = *M*_w_/*M*_n_) is consistently less than 1.10. The numerical value assigned to each polymer identifies its DOS (in mol%). In all cases, these sulfonated block polymers were first dissolved in tetrahydrofuran (THF), and the solutions were then cast into Teflon molds. Following solvent removal, the resultant films were subjected to virucidal testing. The Nafion substrate was obtained in film form from Chemours Company and used as‐received.

**Table 1:** Molecular characteristics of the pre‐sulfonated block polymers employed in this study^a)^

Polymer	*M*_n_ (kDa)	*w*_S_ (wt%)	*w*_T_ (wt%)
TESET	78	36	38
TST	122	66	34
SEBS	56	30	—

^a)^ The S and T subscripts refer to polystyrene and poly(*tert*‐butylstyrene), respectively.

The SARS‐CoV‐2 USA‐WA1/2020 virus described by Harcourt et al.^[^ ^27^ ^]^ was propagated on VeroE6 cells in DMEM with 2% fetal bovine serum (FBS), GlutaMAX, sodium pyruvate, non‐essential amino acids, and antibiotic‐antimycotic. The VeroE6 cells used in these assays were seeded a day prior to the assay at a density of 8 × 10^5^ cells per well in 6‐well plates. Test coupons of film were trimmed to 2 cm^2^ squares and placed into a sterile 6‐well plate. Five drops of 10 µL (50 µL total) of virus were added to the surface of each coupon and exposed for 5, 10, 20, or 30 min. At the end of the exposure period, the coupons were added to 2 mL media (DMEM with 2% FBS and 1× Antibiotic‐Antimycotic). Samples that had been incubated for 30 min were vortexed for 30 s, centrifuged at 1000 RFC for 2 min, and serially diluted. [It was confirmed that vortexing had no discernible effect on the results reported here.] Samples that had been incubated for 5, 10, and 20 min were vortexed for 2–3 s, after which coupons were immediately removed from the media. The eluted media was serial diluted using half‐logarithmic dilutions from 10^0^ to 10^−2.5^. Dilutions were plated in triplicate (200 µL per well) and incubated for 1 h ± 10 min at 37 °C and 5% CO_2_ with periodic rocking. Following the incubation period, wells were overlaid with 2 mL of a 1:1 mixture of 2.5% Avicel RC‐591 (DuPont Nutrition & Health), 2× Temin's Modified Eagle Media with 10% FBS, 2× GlutaMAX, and 2× Antibiotic‐Antimycotic. After a 2‐day incubation, the plates were fixed with 10% neutral buffered formalin, removed from biocontainment, and stained with a 0.2% Gentian Violet and 10% neutral buffered formalin solution. Plaques were counted and used to calculate the titer. The minimum detection level (MDL) was 3 PFU mL^−1^, where PFU refers to a plaque‐forming unit.

The HCoV‐229E virus was propagated on the human hepatocarcinoma cell line (Huh‐7) in cell growth media (DMEM, 1% antibiotics, 10% FBS) at 33 °C. Materials were cut to fit in the bottom of a 96‐well plate, and 25 µL of virus suspension (stock concentration 1.5 × 10^8^ TCID_50_ mL^−1^) was added to the plates for a selected length of time. After exposure, 75 µL of infection media (MEM 1% antibiotics, 1% FBS, 1% HEPES buffer) was added, and the virus was eluted by triturating several times, followed by rapid transfer to new wells. Virus suspensions were immediately diluted serially tenfold from 10^−1^ to 10^−8^. Six replicates of each dilution (50 µL per well) were used to infect Huh‐7 cells seeded the previous day at a density of 10^4^ cells per well. After 2 h, 50 µL of cell growth media was added and the plates were incubated at 33 °C with 5% CO_2_. After 96 h, the cytopathic effect was monitored by visual inspection of the wells. Under the assay conditions employed here, the MDL was determined to be 6.32 × 10^2^ TCID_50_ mL^−1^.

### Statistical Analysis

Corresponding TCID_50_ values were calculated using the Spearman–Kaerber method.^[^ ^28^, ^29^ ^]^ These values correspond to log_10_(50% end‐point dilution) and are calculated from −(*x*_0_ − d/2 + d ∑*_i_* r*_i_*/n*_i_*), where x_0_ corresponds to the log_10_ of the lowest concentration (highest dilution), d is the log_10_ of the dilution factor, r*_i_* represents the number of positive cells in dilution *i*, and n*_i_* is the total number of cells in the *i*th dilution. Other statistical approaches^[^ ^30^, ^31^ ^]^ could likewise have been used for this purpose. After determining the mean and standard deviation/error from the titers as functions of virus, substrate, and exposure time, the statistical significance of each measurement was calculated from the unpaired student's two‐tailed *t*‐test.^[^ ^32^ ^]^ In this case, the *t*‐value was determined from

(1) $$t\;=\;\frac{M_{1}-M_{2}}{S_{p}\sqrt{\left(\frac{1}{n_{1}}+\frac{1}{n_{2}}\right)}}$$

where *M* and *n* in this context denote the mean and replicate number, respectively, the subscripts 1 and 2 represent the control and test specimens, respectively, and *S*_p_ is the pooled standard deviation given by

(2) $$S_{p}=\sqrt{\frac{\left(n_{1}-1\right)S_{1}^{2}+\left(n_{2}-1\right)S_{2}^{2}}{n_{1}+n_{2}-2}}\;$$

Here, S*_i_* is the standard deviation of the *i*th specimen set. The *t*‐value and its corresponding *p*‐value, which provides a measure of statistical significance, can be extracted from tables or, as done here, calculated from an on‐line algorithm.^[^ ^33^ ^]^ Statistical significance is achieved when *p* < 0.05. The results of this analysis are summarized in **Table** **2** and only apply to titers above the MDL.

**Table 2:** Calculated *p*‐values of the antiviral properties afforded by the partially sulfonated block polymers investigated in this study^a)^

	Time [min]			
Virus/specimen designation	5	10	20	30
**SARS‐CoV‐2 virus**				
TESET52	Below MDL	Below MDL	Below MDL	Below MDL
**HCoV‐229E virus**				
TESET26	0.1273	0.0498	0.0458	0.0458
TESET52				
25 °C	0.8133	0.0467	Below MDL	Below MDL
4 °C	0.9187	0.4217	Below MDL	Below MDL
37 °C	0.0498	0.0461	0.0459	0.0458
TST17	1.0000	0.3818	0.2999	0.2130
TST40	0.1273	0.0459	Below MDL	Below MDL
TST63	0.0694	0.0459	Below MDL	Below MDL
SEBS44	0.0495	0.0463	0.0458	0.0458

^a)^ A time point at which a virus titer registers below the MDL is not assigned a *p*‐value if all the observations for that time point lie below the MDL.

## Results and Discussion

**Figure 1:**
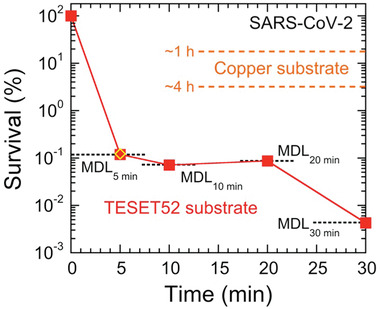
Survival of the SARS‐CoV‐2 virus on the TESET52 anionic block polymer at several exposure times relative to controls performed on a non‐sulfonated block polymer. Results are obtained at minimum detection limits (MDLs, dotted and labeled lines), which reflect differences in the virus stock and control titers (see the text). For comparison, the outcome of the durability test performed 24 h after media exposure on the TESET52 polymer is included (diamond), as are the reported^[^ ^10^ ^]^ survival levels of SARS‐CoV‐2 on copper after ≈1 and 4 h (dashed and labeled lines). Measurements listed at the MDL indicate at or below the corresponding survival level, and error bars represent the standard error. The color‐matched solid line serves to connect the data.

The survival of SARS‐CoV‐2 on TESET52 is presented as a function of exposure time in **Figure** **1** and confirms complete inactivation within experimental detection in 5 min. Included for comparison are results for SARS‐CoV‐2 on copper after 60 and 240 min.^[^ ^10^ ^]^ The MDL corresponds to at least 99.9% inactivation, validating that the TESET52 polymer inactivates SARS‐CoV‐2 over the course of minutes, not hours. Differences in the MDL in Figure 1 reflect variations in the initial virus stock suspension (from 2.15 × 10^3^ PFU/sample at 5 min to 6.65 × 10^4^ PFU/sample at 30 min), which account for marginally different control titers (from 1.78 × 10^3^ to 4.96 × 10^4^ PFU/sample, respectively). As mentioned earlier, no *p*‐values are calculated for these studies (or included in Table 2), as all the corresponding survival results lie below the MDL. The mechanism by which viral inactivation proceeds here is based on a precipitous drop in surface pH (to < 1) due to the presence of sulfonic acid groups on the polymer.^[^ ^23^ ^]^ According to our previous studies, this block polymer self‐organizes into a nanoscale morphology composed of hydrophobic cylinders and alternating lamellae when cast from THF.^[^ ^34^ ^]^ Solvent‐vapor annealing generates a highly ordered lamellar morphology, which represents the equilibrium morphology of this polymer, in agreement with computer simulations.^[^ ^35^ ^]^

**Figure 2:**
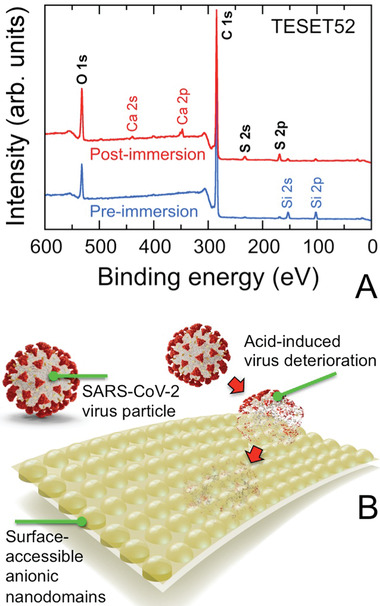
A) XPS spectra collected from the TESET52 polymer before (blue) and after (red) exposure to PBS solution. Ionization peaks relevant to the polymer (discussed in the text and listed in Table 3) are labeled (black). Peaks indicating the presence of Si 2s and 2p are attributed to the glass substrate, whereas those corresponding to Ca 2s and 2p reflect the presence of complexed cations from the PBS solution (the weak Na KLL peak near 500 eV provides additional evidence for complexed cationic species). The peak at ≈400 eV (presumably due to N 1s) is not present in all the spectra collected and is not considered further. B) Stylized illustration of the surface‐reconstructed TESET52 polymer after exposure to moisture or an aqueous medium, indicating that the anionic microdomains needed for pH reduction and SARS‐CoV‐2 inactivation become surface‐accessible.

Since viral inactivation occurs at the polymer/pathogen interface, the surface chemistry of the TESET52 film is particularly important (although we note that the proton reservoir required to sufficiently lower the surface pH depends on the number of contributing sulfonic acid groups and must therefore exceed a minimum film thickness). An X‐ray photoelectron spectroscopy (XPS) spectrum acquired from the surface of an as‐cast TESET52 film is provided in **Figure** **2**A and confirms the existence of ionization peaks for C 1s and O 1s, as expected from Scheme 1. The barely discernible peaks for S indicates that hydrophobic (i.e., low surface energy) lamellae preferentially reside at the surface. Upon exposure to phosphate‐buffered saline (PBS) solution, two additional pairs of peaks become apparent in Figure 2A for Ca in the form of a mobile cation that can complex with sulfonic acid groups, as well as S, which provides chemical evidence of water‐induced surface topological rearrangement (illustrated in Figure 2B). Elemental compositions discerned from XPS spectra such as the ones displayed in Figure 2A are listed for species at or above 1 at% in **Table** **3**. In addition to sulfur, enrichment of oxygen at the surface of the TESET52 film after exposure to PBS solution is consistent with an increase in the population of sulfonic acid groups at the film surface.

**Table 3:** Elemental compositions of TESET52 surfaces before and after immersion in PBS solution at ambient temperature according to XPS analysis^a)^

	Element content [at%]				
	C	O	S	Si	Ca
Before immersion	87.49 ± 0.26	7.91 ± 0.29	0.53 ± 0.03	4.07 ± 0.06	—
After immersion	82.89 ± 0.65	12.31 ± 0.10	2.31 ± 0.26	1.21 ± 0.05	1.15 ± 0.21

^a)^ This compilation only includes elements present at or above 1 at%.

**Figure 3:**
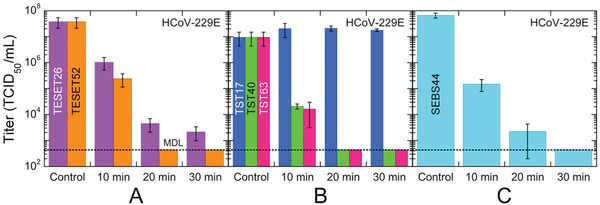
Infectivity of the HCov‐229E virus on three different anionic block polymers—A) TESET, B) TST, and C) SEBS—at several exposure times. The numerical designations assigned to each polymer represent the DOS (in mol%), and the displayed MDL (dotted line) corresponds to 632 TCID_50_ mL^−1^. While most of these data are statistically significant (see the *p*‐values listed in Table 2), those for the TST17 polymer are not. In all cases, measurements listed at the MDL indicate at or below the corresponding titer level, and error bars represent the standard error.

Comparable behavior is evident in **Figure** **3**A for HCoV‐229E on two TESET surfaces differing in DOS. While both materials are virucidal, this outcome indicates that i) the TESET52 polymer with a higher DOS is ultimately more effective at inactivating HCoV‐229E than its TESET26 homolog, and ii) the TESET26 polymer possesses an insufficient DOS to achieve detection limit inactivation over the course of 30 min. As noted earlier, the DOS must be above a critical level for detection‐limit inactivation to be achieved after relatively short exposure times, such as those investigated here. Similar results have been previously reported^[^ ^23^ ^]^ for MRSA. Due to the presence of flexible intermediate blocks, the TESET polymers constitute examples of thermoplastic elastomers and exhibit robust mechanical properties. Removal of these blocks yields the TST block polymers, which are relatively brittle plastics. The response of HCoV‐229E exposed to three TST polymers varying in DOS reveals in Figure 3B that the TST17 polymer has an insufficiently low DOS level and is therefore unable to inactivate the virus, whereas the TST40 and TST63 polymers achieve at least 99.998% inactivation of HCoV‐229E in 20 min. An important consideration regarding midblock‐sulfonated block polymers is that they remain intact in polar solvent and can, at high levels of sorbed water, behave as stable hydrogels because of their nonpolar physical crosslinks.^[^ ^36^, ^37^ ^]^ In marked contrast, if the DOS is sufficiently high, the mechanical properties of endblock‐sulfonated thermoplastic elastomers can be severely compromised upon exposure to polar solvents, which serve to plasticize the physical crosslinks and thus degrade the mechanical stability of the polymer network. With this potential shortcoming in mind, results from HCoV‐229E exposed to an endblock‐sulfonated SEBS block polymer with 44 mol% endblock sulfonation are included in Figure 3C and establish that nearly complete inactivation is likewise achieved in 20 min. It is important to note that, at this DOS level, the polymer behaves as a swollen, solid‐like film in the presence of the virus suspension.

**Figure 4:**
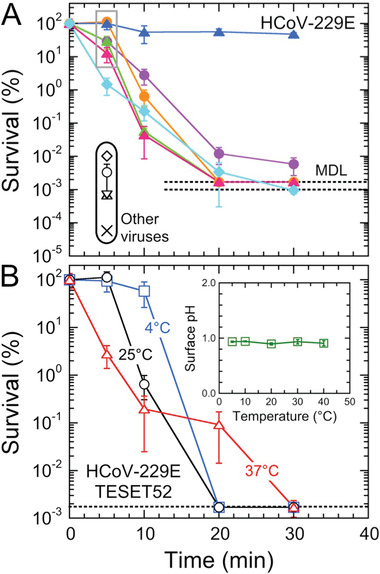
A) Survival of the HCoV‐229E virus on different anionic block polymers (color‐matched to the titer data in Figure 3). The symbols (black open) in the black oval at 5 min correspond to previously examined^[^ ^23^ ^]^ viruses (HAd‐5, circle; influenza A, triangle; VSV, X), as well as two additional ones [PI‐3 (diamond) and X‐MulV (inverted triangle)], on TESET52. The data at 5 min (in the gray box) are statistically non‐significant, according to the *p*‐values listed in Table 2. B) Survival of HCoV‐229E on TESET52 as a function of exposure time at three different temperatures (in °C, labeled, and color‐coded): 4, 25, and 37. The temperature dependence of the surface pH of DI water is included in the inset. In all cases, measurements listed at the MDL (dotted line) indicate at or below the corresponding survival level, and error bars represent the standard error. Color‐matched solid lines serve to connect the data.

Our observations in Figure 3 are compiled in the form of HCoV‐229E survival, defined as the ratio of a titer at a given exposure time normalized relative to that of the stock viral suspension multiplied by 100%, in **Figure** **4**A. Insofar as the DOS level of the styrenic block (whether it is a midblock or an endblock) is sufficiently high, all of the anionic polymers examined here are inherently virucidal, rapidly inactivating both virus strains to their respective MDL: 99.9% for SARS‐CoV‐2 in 5 min and 99.998% for HCoV‐229E in 20 min. These findings suggest that this class of polymeric materials could be equally effective against previous coronavirus outbreaks (e.g., SARS‐CoV‐1 and MERS‐CoV), as well as mutations in the future. To corroborate the comprehensive virucidal nature of the TESET52 polymer in particular, several other viral strains—human adenovirus‐5 (HAd‐5), influenza A virus, vesicular stomatitis virus (VSV), parainfluenza‐3 virus (PI‐3), and xenotropic murine leukemia virus (X‐MulV)—are also displayed in Figure 4A after an exposure time of 5 min. Since the SARS‐CoV‐2^[^ ^11^ ^]^ and HCoV‐229E^[^ ^25^ ^]^ viruses are sensitive to pH and temperature changes, we have further explored the inactivation of HCoV‐229E on TESET52 at three different temperatures in Figure 4B. As the temperature is increased, virus inactivation becomes more pronounced at short times (5 min). At longer times, however, complete inactivation occurs faster at 4 and 25 °C (20 min) than at 37 °C (30 min). Since the surface pH of deionized (DI) water on TESET52 does not change to any appreciable extent with increasing temperature (see the inset in Figure 4B), the latter observation suggests that the pH rises at 37 °C in the presence of virus media due to accelerated sulfonic acid neutralization caused by mobile cations in the media.

The *p*‐values associated with the statistical significance of the titers measured from the SARS‐CoV‐2 and HCoV‐229E viruses presented in Figures 1, 3, and 4 are compiled in Table 2 for those values that lie above the MDL. All the measurements from the SARS‐CoV‐2 analysis in Figure 1 lie below the MDL, but many of the tests focusing on HCoV‐229E yield quantifiable *p*‐values. For example, the titers collected after an exposure time of 5 min from the sulfonated TST and TESET polymers at 25 °C are not statistically significant (1.00 > *p* > 0.07) relative to the stock virus suspension, which we utilize here as the control. To reflect this result, the corresponding survival values are highlighted (boxed) in Figure 4a. Similarly, none of the *p*‐values calculated for the TST17 materials at different exposure times is statistically significant (1.00 > *p* > 0.21) with respect to the stock virus suspension. Since differences between the stock suspension and titers measured from the unsulfonated copolymers are also not statistically significant (0.50 > *p* > 0.06), use of the stock suspension as the normalizing value for the TST and TESET polymers in Figure 4 and the control in *p*‐value calculations ensures consistency of analysis, as well as a single value of the MDL for, and systematic comparison of, all the materials in these two series. Interestingly, Table 2 furthermore reveals that, at all exposure times examined from 5 to 30 min, the titers measured for the SEBS44 polymer at 25 °C and the TESET52 polymer at 37 °C are statistically significant (*p* < 0.05), whereas those for the TESET52 polymer at 4 °C are not at short times.

Unlike one‐time‐use disinfectants that require repeated application at discrete times to inactivate infectious microbes without providing future prevention of (re)contamination, the antimicrobial properties of the materials examined here are durable and remain antiviral after use, as demonstrated by the inactivation of SARS‐CoV‐2 on TESET52. In an independent test, polymer coupons were submerged in media (DMEM with 2% FBS and 1× Antibiotic‐Antimycotic) and quickly removed after gentle swirling (without vortexing) so that the exposure time was only a few seconds. After a drying time of 24 h, analysis of SARS‐CoV‐2 yielded the following titers after exposure for 5 min: 2.56 × 10^3^ PFU/sample stock suspension, 2.46 × 10^3^ PFU/sample control and undetectable virus after exposure (at or below the MDL). These results are included in Figure 1 and confirm the continuous efficacy of the TESET52 polymer. Since the inactivation mechanism depends sensitively on the surface pH of these anionic block polymers, analysis of surface pH provides a predictive measure of inactivation performance. As reported elsewhere,^[^ ^23^ ^]^ for example, these anionic block polymers not only remain continuously antimicrobial (until the sulfonic acid groups are appreciably neutralized) but can also be subsequently recharged to full effectiveness for further use by short‐time (< 1 h) exposure to relatively dilute aqueous acids. Moreover, the pH of TESET52 remains unaffected after it is initially exposed to DI water (pH = 0.96), dried for 5 days, and re‐exposed to DI water (pH = 0.99), indicating that the polymer, if activated by a relatively cation‐free water source, would remain effective after at least several exposures to virus.

The results presented in Figures 1, 3, and 4 altogether confirm that the nanostructured polymers investigated here are highly effective against both SARS‐CoV‐2 and HCoV‐229E, in addition to other bacteria and viruses reported earlier.^[^ ^23^ ^]^ While numerous polymeric and organic/inorganic hybrid materials have been described as antimicrobial, most are actually not comprehensive and only provide antibacterial properties. Recent reviews, however, have specifically addressed the development of polymers as antiviral media in the food^[^ ^38^ ^]^ and health^[^ ^39^ ^]^ sectors. Here, we examine several antiviral polymer designs and demonstrate that the sulfonated materials considered in the present study are ideally suited to combat human coronaviruses by affording significantly more expedient antiviral properties. Diblock copolymers containing poly(acrylic acid), for example, have been observed^[^ ^40^ ^]^ to be highly effective against both Gram‐positive and Gram‐negative bacteria (not viruses), and require exposure times on the order of several hours. Although some materials are more broadly suitable for antibacterial and antifungal (not antiviral) applications,^[^ ^41^ ^]^ they do not rely on a pH‐drop afforded by the presence of acidic moieties. Instead, Guo et al.^[^ ^42^ ^]^ use such groups for metal‐cation binding purposes only. Early studies by Feltz and Regelson^[^ ^43^ ^]^ confirm an interest in ethylene/maleic anhydride copolymers as antiviral materials, but their results involving the Echo 9 virus on just the copolymer indicate little, if any, antiviral activity. Although Mengo virus is believed^[^ ^44^ ^]^ to be resistant to anionic polymers, Merigan and Finkelstein^[^ ^45^ ^]^ have reported that several viruses including Mengo virus can be inactivated (up to 94%) by a divinyl ether/maleic anhydride copolymer after exposure for 1 h at 38 °C. Other polycarboxylates containing maleic or acrylic acid units have been found^[^ ^46^ ^]^ to be incapable of inactivating tobacco mosaic virus, whereas a styrene‐*alt*‐maleic acid copolymer could completely inactivate human immunodeficiency virus (HIV) after it was incorporated into mammalian cells but not before.^[^ ^47^ ^]^ In contrast, poly(vinyl alcohol sulfate) and its acrylic acid copolymer inhibit the growth of HIV, but remain ineffective against a wide range of other viruses including influenza.^[^ ^48^ ^]^ Numerous studies have examined the general benefit of acidic polymers to prevent virus proliferation for in vivo applications^[^ ^49^, ^50^, ^51^, ^52^, ^53^ ^]^ that require substantially longer exposure times than those examined here for surface disinfection. To the best of our knowledge, only the present anionic polymers with sufficiently robust mechanical properties have enabled use of a surface pH‐drop mechanism to inactivate human coronaviruses, as well as other bacteria and viruses, on fomites for the purpose of preventing disease transmission.

**Figure 5:**
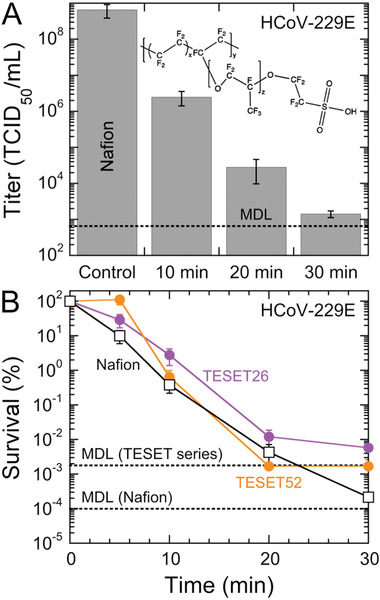
A) Infectivity of the HCov‐229E virus on Nafion at several exposure times. Included is the chemical structure of Nafion illustrating its highly fluorinated (and, hence, hydrophobic) content, as well as its hydrophilic sulfonic acid moiety. Associated *p*‐values range from ≈0.07 to ≈0.09. The displayed MDL (dotted line) corresponds to 632 TCID_50_ mL^−1^. B) Comparison of the survival of the HCoV‐229E virus on the Nafion and TESET polymers (labeled and color‐coded). In all cases, measurements listed at the MDL indicate at or below the corresponding titer level. Color‐matched solid lines serve to connect the data, and error bars represent the standard error in both (A) and (B).

Thus far, we have only considered antiviral polymers on the basis of one archetype of nanostructured amphiphilic polymers derived from microphase‐separated block polymers. Another polymer exhibiting comparable water sorption and diffusion properties^[^ ^54^ ^]^ as the TESET52 polymer is Nafion, the chemical structure of which is provided in **Figure** **5**a. While Nafion is a relatively expensive polymer that is most closely associated with proton‐exchange membrane fuel cells,^[^ ^55^ ^]^ both materials have been successfully used in technologies requiring high water solubility and transmission, such as ionic polymer‐metal composites as electroactive media^[^ ^56^ ^]^ and gas‐separation membranes for CO_2_ removal/capture.^[^ ^57^ ^]^ At nanoscale dimensions, both materials exhibit continuous hydrophilic pathways, although those in Nafion are significantly smaller (just a few nanometers across, according to cryoelectron tomography^[^ ^58^ ^]^) than those formed in the TESET polymers, but they can be selectively swollen by incorporating a hydrophilic (e.g., ionic^[^ ^59^ ^]^) liquid. Included in Figure 5a are HCoV‐229E titers measured at different exposure times. These results appear qualitatively similar to those displayed for the TESET and TST polymers, revealing that this anionic polymer likewise possesses antiviral properties in film form. We note here, however, that the *p*‐values for these measurements lie just outside the criterion established here for statistical significance (i.e., *p* < 0.05). For completeness, the *p*‐values for HCoV‐229E on Nafion range from 0.07 to 0.09 and thus lie within the 90%, rather than 95%, confidence interval. Normalized survival values are presented for the TESET and Nafion series in Figure 5b and confirm that all these nanostructured amphiphilic polymers are similarly virucidal, although the TESET52 polymer is the only one that reaches the MDL within an exposure time of 30 min. It is interesting that both Nafion and the TESET26 polymer possess comparable sulfonate levels corresponding to ion‐exchange capacities (IECs) of ≈1, whereas the IEC of the TESET52 polymer is 2.

## Conclusions

In summary, this study introduces a largely unexplored preventative coating strategy that relies on the use of nanostructured anionic polymers to generate low surface pH levels (due to the presence of protons from sulfonic acid groups that become surface accessible upon exposure to an aqueous medium) to expediently inactivate coronaviruses. The precipitous pH drop afforded by these substrates promotes rapid and repetitive viral inactivation to i) reduce the likelihood of human coronavirus transmission due to contact with contaminated surfaces and ii) mitigate the spread of SARS‐CoV‐2 in particular, as well as other infectious pathogens in general. Since this self‐disinfecting strategy does not target specific chemical moieties on microbes, it is non‐specific and should not result in the development of microbial resistance, which is not necessarily the case for recent metal‐oxide approaches^[^ ^60^ ^]^ that likewise show promise in combating coronavirus. Moreover, the comprehensive antimicrobial nature of this class of polymeric materials makes them particularly suitable for applications in which microbes form protective spores (e.g., Clostridioides difficile,^[^ ^61^ ^]^ a highly contagious anaerobic pathogen that can cause fatal pseudomembranous colitis) or a plethora of infectious microbes coexist to synergistically promote convoluted health problems. Last, these polymers do not introduce known negative health or environmental implications, and the anionic block polymers can be recycled to avoid adding solid waste to landfills and natural resources.

## Conflict of Interest

The authors declare no conflict of interest.

## Data Availability

Research data are not shared.

## References

[1] M. Nicola , Z. Alsafi , C. Sohrabi , A. Kerwan , A. Al‐Jabir , C. Iosifidis , M. Agha , R. Agha , Int. J. Surg. 2020, 78, 185. 32305533 10.1016/j.ijsu.2020.04.018 PMC7162753

[2] Wilder‐Smith , D. O. Freedman , J. Travel Med. 2020, 27, taaa020. 32052841 10.1093/jtm/taaa020 PMC7107565

[3] https://coronavirus.jhu.edu/map.html (Accessed: February 2021).

[4] Q.‐Y. Peng , X.‐T. Wang , L.‐N. Zhang , Intensive Care Med. 2020, 46, 849. 32166346 10.1007/s00134-020-05996-6 PMC7080149

[5] M. Madjid , P. Safavi‐Naeini , S. D. Solomon , O. Vardeny , JAMA, J. Am. Med. Assoc. 2020, 5, 831. 10.1001/jamacardio.2020.1286 32219363

[6] L. Verdoni , A. Mazza , A. Gervasoni , L. Martelli , M. Ruggeri , M. Ciuffreda , E. Bonanomi , L. D'Antiga , Lancet 2020, 395, 1771. 32410760 10.1016/S0140-6736(20)31103-X PMC7220177

[7] M. A. Shereen , S. Khan , A. Kazmi , N. Bashir , R. Siddique , J. Adv. Res. 2020, 24, 91. 32257431 10.1016/j.jare.2020.03.005 PMC7113610

[8] C. R. MacIntyre , A. A. Chughtai , BMJ 2015, 350, h694. 25858901 10.1136/bmj.h694

[9] J. Cai , W. Sun , J. Huang , M. Gamber , J. Wu , G. He , Emerging Infect. Dis. 2020, 26, 1343. 10.3201/eid2606.200412 PMC7258486 32163030

[10] N. van Doremalen , T. Bushmaker , D. H. Morris , M. G. Holbrook , A. Gamble , B. N. Williamson , A. Tamin , J. L. Harcourt , N. J. Thornburg , S. I. Gerber , J. O. Lloyd‐Smith , E. de Wit , V. J. Munster , N. Engl. J. Med. 2020, 382, 1564. 32182409 10.1056/NEJMc2004973 PMC7121658

[11] A. W. H. Chin , J. T. S. Chu , M. R. A. Perera , K. P. Y. Hui , H.‐L. Yen , M. C. W. Chan , M. Peiris , L. L. M. Poon , Lancet Microbe 2020, 1, e10. 32835322 10.1016/S2666-5247(20)30003-3 PMC7214863

[12] S. Riddell , S. Goldie , A. Hill , D. Eagles , T. W. Drew , Virol. J. 2020, 17, 145. 33028356 10.1186/s12985-020-01418-7 PMC7538848

[13] L. Casanova , E. Alfano‐Sobsey , W. A. Rutala , D. J. Weber , M. Sobsey , Emerging Infect. Dis. 2008, 14, 1291. 10.3201/eid1408.080085 PMC2600382 18680659

[14] https://www.bbc.com/news/health‐53325771 (Accessed: September 2020).

[15] P. I. Hora , S. G. Pati , P. J. McNamara , W. A. Arnold , Environ. Sci. Technol. Lett. 2020, 7, 622. 10.1021/acs.estlett.0c00437 37566314

[16] A. P. Richter , J. S. Brown , B. Bharti , A. Wang , S. Gangwal , K. Houck , E. A. Cohen Hubal , V. N. Paunov , S. D. Stoyanov , O. D. Velev , Nat. Nanotechnol. 2015, 10, 817. 26167765 10.1038/nnano.2015.141

[17] V. B. Schwartz , F. Thétiot , S. Ritz , S. Pütz , L. Choritz , A. Lappas , R. Förch , K. Landfester , U. Jonas , Adv. Funct. Mater. 2012, 22, 2376.

[18] J. A. Lemire , J. J. Harrison , R. J. Turner , Nat. Rev. Microbiol. 2013, 11, 371. 23669886 10.1038/nrmicro3028

[19] M. C. Stensberg , Q. Wei , E. S. McLamore , D. M. Porterfield , A. Wei , M. S. Sepúlveda , Nanomedicine 2011, 6, 879. 21793678 10.2217/nnm.11.78 PMC3359871

[20] Y. Pan , Q. Xia , H. Xiao , Polymers 2019, 11, 1283. 10.3390/polym11081283 PMC6723773 31374864

[21] B. S. T. Peddinti , F. Scholle , R. A. Ghiladi , R. J. Spontak , ACS Appl. Mater. Interfaces 2018, 10, 25955. 30044089 10.1021/acsami.8b09139

[22] B. S. T. Peddinti , N. Morales‐Gagnon , B. Pourdeyhimi , F. Scholle , R. J. Spontak , R. A. Ghiladi , ACS Appl. Mater. Interfaces 2021, 13, 155. 33356100 10.1021/acsami.0c16953

[23] B. S. T. Peddinti , F. Scholle , M. G. Vargas , S. D. Smith , R. A. Ghiladi , R. J. Spontak , Mater. Horiz. 2019, 6, 2056.

[24] C. M. Bates , F. S. Bates , Macromolecules 2017, 50, 3.

[25] A. Lamarre , P. J. Talbot , Can. J. Microbiol. 1989, 35, 972. 2819602 10.1139/m89-160

[26] G. Kampf , D. Todt , S. Pfaender , E. Steinmann , J. Hosp. Infect. 2020, 104, 246. 32035997 10.1016/j.jhin.2020.01.022 PMC7132493

[27] J. Harcourt , A. Tamin , X. Lu , S. Kamili , S. K. Sakthivel , J. Murray , K. Queen , Y. Tao , C. R. Paden , J. Zhang , Y. Li , A. Uehara , H. Wang , C. Goldsmith , H. A. Bullock , L. Wang , B. Whitaker , B. Lynch , R. Gautam , C. Schindewolf , K. G. Lokugamage , D. Scharton , J. A. Plante , D. Mirchandani , S. G. Widen , K. Narayanan , S. Makino , T. G. Ksiazek , K. S. Plante , S. C. Weaver , S. Lindstrom , S. Tong , V. D. Menachery , N. J. Thornburg , Emerging Infect. Dis. 2020, 26, 1266. 10.3201/eid2606.200516 PMC7258473 32160149

[28] C. Spearman , Br. J. Psychol. 1908, 2, 227.

[29] G. Kaerber , Naunyn‐Schmiedeberg's Arch. Exp. Pathol. Pharmacol. 1931, 162, 480.

[30] L. J. Reed , H. Muench , Am. J. Epidemiol. 1938, 27, 493.

[31] M. A. Ramakrishnan , World J. Virol. 2016, 5, 85. 27175354 10.5501/wjv.v5.i2.85 PMC4861875

[32] S. Boslaugh , Statistics in a Nutshell, O'Reilly Media, Sebastopol, CA 2012.

[33] https://www.socscistatistics.com/tests/studentttest/default2.aspx (Accessed: September 2020).

[34] K. P. Mineart , X. Jiang , H. Jinnai , A. Takahara , R. J. Spontak , Macromol. Rapid Commun. 2015, 36, 432. 25537368 10.1002/marc.201400627

[35] K. P. Mineart , B. Lee , R. J. Spontak , Macromolecules 2016, 49, 3126.

[36] K. P. Mineart , H. A. Al‐Mohsin , B. Lee , R. J. Spontak , Appl. Phys. Lett. 2016, 108, 101907.

[37] K. P. Mineart , J. D. Dickerson , D. M. Love , B. Lee , X. Zuo , R. J. Spontak , Macromol. Rapid Commun. 2017, 38, 1600666. 10.1002/marc.201600666 28117518

[38] W. Randazzo , M. J. Fabra , I. Falcó , A. López‐Rubio , G. Sánchez , Compr. Rev. Food Sci. Food Saf. 2018, 17, 754. 33350126 10.1111/1541-4337.12349

[39] N. Jarach , H. Dodiuk , S. Kenig , Polymers 2020, 12, 1727. 10.3390/polym12081727 PMC7464166 32752109

[40] G. Gratzl , C. Paulik , S. Hild , J. P. Guggenbichler , M. Lackner , Mater. Sci. Eng., C 2014, 38, 94. 10.1016/j.msec.2014.01.050 24656357

[41] X. He , Y. Yang , H. Song , S. Wang , H. Zhao , D. Wei , ACS Appl. Mater. Interfaces 2020, 12, 14784. 32141282 10.1021/acsami.9b20733

[42] J. Guo , Q. Xu , R. Shi , Z. Zheng , H. Mao , F. Yan , Langmuir 2017, 33, 4346. 28388842 10.1021/acs.langmuir.7b00185

[43] E. T. Feltz , W. Regelson , Nature 1962, 196, 642. 13962902 10.1038/196642a0

[44] J. B. Campbell , J. S. Colter , Can. J. Microbiol. 1967, 13, 931. 4292852 10.1139/m67-125

[45] T. C. Merigan , M. S. Finkelstein , Virology 1968, 35, 363. 4298648 10.1016/0042-6822(68)90215-8

[46] A. Stein , G. Loebenstein , Phytopathology 1972, 62, 1461.

[47] V. Pirrone , S. Passic , B. Wigdahl , R. F. Rando , M. Labib , F. C. Krebs , BioMed Res. Int. 2010, 2010, 548749. 10.1155/2010/548749 PMC2879553 20589074

[48] D. Schols , E. De Clercq , J. Balzarini , M. Baba , M. Witvrouw , M. Hosoya , G. Andrei , R. Snoeck , J. Neyts , R. Pauwels , M. Nagy , J. Györgyi‐Edelényi , R. Machovich , I. Horváth , M. Low , S. Görög , Antiviral Chem. Chemother. 1990, 1, 233.

[49] B. Helbig , R. Klöcking , P. Wutzler , Antiviral Chem. Chemother. 1997, 8, 265. 10.1177/095632020201300405 12495212

[50] A. R. Neurath , N. Strick , Y.‐Y. Li , BMC Infect. Dis. 2002, 2, 27. 12445331 10.1186/1471-2334-2-27 PMC139971

[51] E. Arnáiz , E. Vacas‐Córdoba , M. Galán , M. Pion , R. Gómez , M. Á. Muñoz‐Fernández , F. J. de la Mata , J. Polym. Sci., Part A: Polym. Chem. 2014, 52, 1099.

[52] A. Vaillant , Antiviral Res. 2016, 133, 32. 27400989 10.1016/j.antiviral.2016.07.004

[53] R. H. Bianculli , J. D. Mase , M. D. Schulz , Macromolecules 2020, 53, 9158.

[54] L. Ansaloni , Z. Dai , J. J. Ryan , K. P. Mineart , Q. Yu , K. T. Saud , M.‐B. Hägg , R. J. Spontak , L. Deng , Adv. Mater. Interfaces 2017, 4, 1700854.

[55] K. A. Mauritz , R. B. Moore , Chem. Rev. 2004, 104, 4535. 15669162 10.1021/cr0207123

[56] P. H. Vargantwar , K. E. Roskov , T. K. Ghosh , R. J. Spontak , Macromol. Rapid Commun. 2012, 33, 61. 22105960 10.1002/marc.201100535

[57] Z. Dai , J. Deng , H. Aboukeila , J. Yan , L. Ansaloni , K. P. Mineart , M. Giacinti Baschetti , R. J. Spontak , L. Deng , NPG Asia Mater 2019, 11, 53.

[58] F. I. Allen , L. R. Comolli , A. Kusoglu , M. A. Modestino , A. M. Minor , A. Z. Weber , ACS Macro Lett. 2015, 4, 1. 35596390 10.1021/mz500606h

[59] Z. Dai , L. Ansaloni , J. J. Ryan , R. J. Spontak , L. Deng , Green Chem. 2018, 20, 1391.

[60] S. Behzadinasab , A. Chin , M. Hosseini , L. Poon , W. A. Ducker , ACS Appl. Mater. Interfaces 2020, 12, 34723. 32657566 10.1021/acsami.0c11425 PMC7385996

[61] Y. Ciftci , B. S. T. Peddinti , R. A. Ghiladi , R. J. Spontak , unpublished.

